# Design and Development of a Mobile Health (mHealth) Platform for Dementia Prevention in the Prevention of Dementia by Mobile Phone Applications (PRODEMOS) Project

**DOI:** 10.3389/fneur.2021.733878

**Published:** 2021-12-16

**Authors:** Melanie Hafdi, Esmé Eggink, Marieke P. Hoevenaar-Blom, M. Patrick Witvliet, Sandrine Andrieu, Linda Barnes, Carol Brayne, Rachael Brooks, Nicola Coley, Jean Georges, Abraham van der Groep, Harm van Marwijk, Mark van der Meijden, Libin Song, Manshu Song, Youxin Wang, Wenzhi Wang, Wei Wang, Anders Wimo, Xiaoyan Ye, Eric P. Moll van Charante, Edo Richard

**Affiliations:** ^1^Department of Neurology, Amsterdam University Medical Center (UMC) Location Academic Medical Center (AMC), Amsterdam, Netherlands; ^2^Department of General Practice, Amsterdam Public Health Research Institute, Amsterdam University Medical Center (UMC), University of Amsterdam, Amsterdam, Netherlands; ^3^Department of Public and Occupational Health, Amsterdam Public Health Research Institute, Amsterdam University Medical Center (UMC), University of Amsterdam, Amsterdam, Netherlands; ^4^Center for Epidemiology and Research in Population Health (CERPOP), University of Toulouse-INSERM UMR1295, Toulouse, France; ^5^Department of Epidemiology and Public Health, Toulouse University Hospital, Toulouse, France; ^6^Cambridge Public Health, University of Cambridge, Cambridge, United Kingdom; ^7^Alzheimer Europe, Luxembourg, Luxembourg; ^8^Philips VitalHealth, Ede, Netherlands; ^9^Department of Primary Care and Public Health, Brighton and Sussex Medical School, Brighton, United Kingdom; ^10^Fuzhou Comvee Network &Technology Co., Ltd, Fuzhou, China; ^11^Beijing Key Laboratory of Clinical Epidemiology, School of Public Health, Capital Medical University, Beijing, China; ^12^School of Medical and Health Sciences, Edith Cowan University, Perth, WA, Australia; ^13^Department of Neuroepidemiology, Beijing Neurosurgical Institute, Beijing, China; ^14^School of Public Health, Shandong First Medical University & Shandong Academy of Medical Sciences, Tai'an, China; ^15^Centre for Precision Health, Edith Cowan University, Perth, WA, Australia; ^16^Division of Neurogeriatrics, Department of Neurobiology, Care Sciences and Society, Karolinska Institutet, Stockholm, Sweden; ^17^Department of Neurology, Radboud University Donders Institute for Brain, Cognition and Behaviour, Nijmegen, Netherlands

**Keywords:** mHealth, dementia, cardiovascular disease(s), prevention, design concept, behavioral health

## Abstract

**Background:** Mobile health (mHealth) has the potential to bring preventive healthcare within reach of populations with limited access to preventive services, by delivering personalized support at low cost. Although numerous mHealth interventions are available, very few have been developed following an evidence-based rationale or have been tested for efficacy. This article describes the systematic development of a coach-supported mHealth application to improve healthy lifestyles for the prevention of dementia and cardiovascular disease in the United Kingdom (UK) and China.

**Methods:** Development of the Prevention of Dementia by Mobile Phone applications (PRODEMOS) platform built upon the experiences with the Healthy Aging Through Internet Counseling in the Elderly (HATICE) eHealth platform. In the *conceptualization* phase, experiences from the HATICE trial and needs and wishes of the PRODEMOS target population were assessed through semi-structured interviews and focus group sessions. *Initial technical development* of the platform was based on these findings and took place in consecutive sprint sessions. Finally, during the *evaluation and adaptation* phase, functionality and usability of the platform were evaluated during pilot studies in UK and China.

**Results:** The PRODEMOS mHealth platform facilitates self-management of a healthy lifestyle by goal setting, progress monitoring, and educational materials on healthy lifestyles. Participants receive remote coaching through a chat functionality. Based on lessons learned from the HATICE study and end-users, we made the intervention easy-to-use and included features to personalize the intervention. Following the pilot studies, in which in total 77 people used the mobile application for 6 weeks, the application was made more intuitive, and we improved its functionalities.

**Conclusion:** Early involvement of end-users in the development process and during evaluation phases improved acceptability of the mHealth intervention. The actual use and usability of the PRODEMOS intervention will be assessed during the ongoing PRODEMOS randomized controlled trial, taking a dual focus on effectiveness and implementation outcomes.

## Introduction

The projected worldwide increase in dementia prevalence is expected to largely occur in low- and middle-income countries and amongst hard-to-reach populations in high-income countries (1, 2). An estimated 30–40% of late-life dementia appears to be attributable to potentially modifiable risk factors, including smoking, insufficient physical activity, and unhealthy diet (3). Interventions targeting these risk factors may have the potential to delay or prevent dementia onset and could be especially beneficial for vulnerable populations, given their high exposure to high risk of these behaviors (4, 5).

The rapid increase of internet access through mobile devices may have the potential to bring preventive healthcare within reach of large groups of people who have limited access to preventive services (6). Mobile health (mHealth) applications can contribute to personalized care and remote delivery of health messaging and services, at low cost and on a global scale (7, 8). Seizing the business opportunity healthcare applications have mushroomed, rising to over 90 000 in app stores in the first quarter of 2020 (9, 10). However, very few of these have been developed following an evidence-based rationale, or have been tested for efficacy in a (randomized controlled) trial. While the conceptualization and architecture of such mHealth interventions are key aspects of development with respect to its perceived usability, uptake, and ultimately success, guidelines to design mHealth interventions for vulnerable populations are not readily available (11).

In the Prevention of Dementia using Mobile Phone Applications (PRODEMOS) trial, we will assess the effectiveness and implementation of a coach-supported mHealth platform to reduce dementia risk over a period of 18 months. The study population will consist of 1,200 older adults with low socioeconomic status (SES) from the United Kingdom (UK) and 1,200 older adults from Beijing, China, all with 2 or more lifestyle factors at levels associated to an increased dementia risk (12). In this article, we describe the development of the PRODEMOS mHealth intervention, from general idea to platform design, and from prototype to pilot study. We make specific recommendations on mHealth design for vulnerable populations, based on extensive interactions with the target population and other important stakeholders, including health care professionals, software developers, and researchers.

## Methods

### Context of PRODEMOS Study

The platform described in this paper was designed as part of the PRODEMOS trial. Development of the PRODEMOS platform built on the Healthy Aging Through Internet Counseling in the Elderly (HATICE) eHealth platform, which was designed and piloted between 2013 and 2016 and proven effective for lowering cardiovascular risk amongst European older adults in a randomized controlled trial (RCT) (13, 14). The coach-supported HATICE platform enabled self-management of cardiovascular risk factors, integrating European guideline recommendations on prevention of cardiovascular disease (CVD) and principles of Bandura's social-cognitive theory of self-management and behavioral change (15).

In PRODEMOS, we will focus on dementia prevention, however, with up to 50% of modifiable risk factors for dementia being cardiovascular risk factors we were still able to incorporate experiences and evidence from the HATICE trial (3, 16, 17). Given the rising smartphone penetration rates worldwide (18), and because especially in LMIC people tend to access and use the internet through smartphones rather than personal computers (19), we decided to develop the PRODEMOS platform as an mHealth intervention. The PRODEMOS platform is built to facilitate the self-management of risk factors for dementia, including overweight, hypertension, high cholesterol, diabetes, unhealthy diet, smoking, and insufficient physical activity. In line with the HATICE platform, PRODEMOS participants are able to set SMART (Specific, Measurable, Achievable, Realistic, Timely) lifestyle goals, enter measurements, read goal-related education materials, and receive personalized lifestyle- and goal setting support via chat messaging from a remote coach.

**Figure 1:**
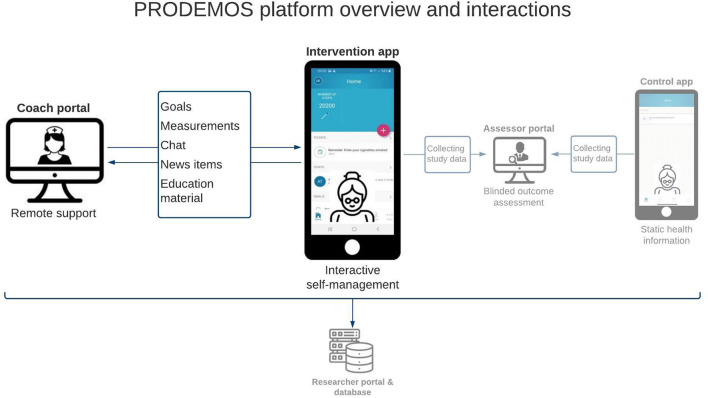
Overview of the PRODEMOS platform and its interactions.

The mobile application will be connected to a coach portal, allowing for remote lifestyle support by a health coach. The PRODEMOS platform also comprises a separate assessor- and researcher portal for data collection and outcome assessment, and a static mobile application with written healthcare advice only and without interactive features, for those randomized to the control condition of the trial. The assessor- and researcher portals and control application have been designed within the research context of the PRODEMOS project, of which the protocol is described in more detail elsewhere (12). Figure 1 shows the components of the PRODEMOS platform and their interrelationships. All key functionalities of the PRODEMOS platform will be similar in the UK and China. Besides differences in language, certain cultural adaptations will be made to ensure adequate fit of the intervention with the target population. The PRODEMOS mobile application will be built to support participants with limited digital literacy, operationalized as at least being able to send a message using a smartphone.

### Phases of Development

**Figure 2:**
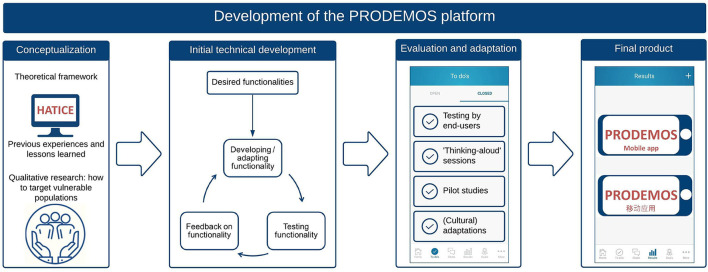
Phases of development of the PRODEMOS platform.

The development of the PRODEMOS platform is visualized in Figure 2. Although technical interventions are typically developed in an iterative cycle of overlapping phases, several distinct phases can be distinguished in the development of the PRODEMOS platform.

#### Conceptualization

First, we performed a thorough evaluation of the HATICE platform, focusing on the perceived value and usability of the eHealth intervention, as well as on the overall implementation. Through thematic analysis of semi-structured interviews with HATICE participants, we learned which factors affected initial and sustained engagement with the eHealth platform (20). In subsequent focus groups, we asked HATICE participants and coaches to share their experiences, views and recommendations for future use of the platform and coach support.

Following this, we assessed the specific needs and wishes of the PRODEMOS target population regarding an mHealth intervention to change their lifestyle behavior. We performed semi-structured interviews with 19 low SES Dutch older adults and 26 Chinese older adults and thematically analyzed them (21). To gather further data on the needs of the target population for successful use of the platform and remote coaching, focus group sessions with older adults of low SES were held in both the UK and the Netherlands. In separate sessions, other stakeholders, including Clinical Research Network nurses and experienced health coaches, were interviewed about their perspectives regarding coach-support for vulnerable populations.

#### Initial Technical Development

Based on the HATICE eHealth platform and the additional lessons learned, the study group drafted an outline capturing all necessary functionalities for the new portal and mobile application. Initial technical development was undertaken by Philips Vital Health (PVH; for the UK) and Fuzhou Comvee Network & Technology (Comvee; for China) in 2-weekly “sprint” sessions over 4–6 months, according to the agile principle (22). In iterative cycles, researchers from the coordinating research team at Amsterdam UMC provided detailed descriptions of all desired functionalities and gave feedback on functionalities that were newly developed.

#### Evaluation and Adaptation

Following initial technical development, the functionality of the portal and mobile application were meticulously evaluated. Software experts from PVH and Comvee and researchers from the coordinating research team, UK, and China internally tested the software. During “thinking aloud” sessions, we asked potential participants to navigate through the mobile application and directly share their thoughts with the developers. The developers also tested user experience (i.e., how the participants interact with the mobile application) and the user interface (i.e., the look and feel, presentation, and interactivity of the mobile application) with potential participants using predefined scripts and success criteria for participants to navigate through the most important functionalities of the application. The functionality of the portal and mobile application was subsequently trialed in six-week pilot studies in the UK and China. We used qualitative data, gathered through focus groups with pilot participants and participating coaches, and data on user statistics to evaluate usability. User statistics included details on goals, measurements, and chat history and were gathered manually from the platform, as the automated export functionality for user statistics had not been finalized by that stage. Findings from the internal test sessions, thinking aloud session, and pilot evaluation informed the final adaptation phase, in which the portal and mobile application was prepared for use in the full trial.

Unless stated otherwise, all qualitative sessions were led by at least one member of the research team and one member of the technical team, following a topic guide. We audiotaped all sessions and shared written summaries with the coordinating research team. Through plenary discussions between the researchers and technical developers, we translated the evaluation results into concrete development steps when deemed appropriate and feasible. More detail on demographics, methodology and recruitment of the evaluation processes is provided in Supplementary Table 1.

## Results

### Conceptualization Phase

#### Lessons Learned From the HATICE Study

Prior to the start of the development of the PRODEMOS mHealth portal and mobile application construction, a qualitative evaluation among participants and coaches of the HATICE intervention took place. This demonstrated that most participants had appreciated the HATICE platform and coach support, and felt that it had helped them to pursue their lifestyle goals. Participants had used the platform mostly in a reactive way, by responding to notifications about chat messages and questionnaires (20). To capitalize on this finding, more (automatic) reminders to enter measurements were built in to the PRODEMOS mobile application, the frequency and content of which can be adjusted to the participant's needs. The qualitative evaluation of HATICE also revealed that participants had a wish for more tailored and frequently updated education material to stimulate sustained engagement over time. Furthermore, they expressed a need for more options to tailor the intervention to (changes in) their personal situation. As a response, we developed several additional features to facilitate personalization of the intervention, as displayed in Supplementary Table 2. Some HATICE participants noted that they had rarely used several functionalities of the intervention and thought that additional guidance, e.g., by adding a tutorial video on the home page, could help participants to make more use of all features of the platform. We therefore built an explanatory animation video accessible through the library of the mobile application, covering the main functionalities of the PRODEMOS application.

From the HATICE trial, we learned that coach support was very important to stimulate both initial and sustained platform use. Participants expressed a need for active encouragement from the coach when a goal was reached or when their commitment weakened. Similarly, as HATICE coaches would have liked to keep better track of their participants' progress, we redesigned certain functionalities of the coach portal to facilitate better support of participants, as shown in Supplementary Table 2.

#### Lessons Learned From Potential PRODEMOS End-Users

Input from focus group sessions and individual interviews with older adults at increased cardiovascular risk in China and of additional low SES in the Netherlands and the UK, and focus groups with healthcare professionals in the UK and China was used to tailor the intervention to the PRODEMOS end-users. For the current section, we distinguish aspects of user-friendliness and personalization of the PRODEMOS mobile application.

##### User-Friendliness

As previously mentioned by the HATICE participants, members of the target population expressed the desire for a simple and intuitive-to-use mobile application, for example as suggested by a 77 year old male interview participant: “*If you're going to introduce this [app], you'd really have to educate a group of people, like how do you use something like that?.”* Both potential coaches and participants favored pre-set options for lifestyle goals, to ensure easy-to-achieve and feasible goals. We developed the goal-setting flow in such a way that participants are able to build their lifestyle goals in several consecutive steps with wide choice from pre-set options, using the SMART principle (e.g., losing weight by increasing physical activity levels by walking twice a week for 30 mins). Participants also indicated the need for positive framing (e.g., ‘improving blood pressure’ as opposed to ‘working on high blood pressure’) and easy (non-medical) language, for example a 63 year old female interview participant: “*[The app has to be] understandable! Don't go tossing around big words and medical terms and all that.”* For this, we have adapted the wording throughout the mobile application. Another important aspect of a user-friendly intervention was trustworthy and easy-to-understand material. Lastly, we have simplified log-in procedures, to facilitate easy access (Supplementary Table 2). Based on wishes from the Chinese target population, we made the Chinese mobile application available as a WeChat sub-application or “mini-program.” WeChat is a widely used Chinese multi-purpose messaging, social media, and mobile payment application with a wide range of such mini-programs.

##### Personalization

In addition to user-friendliness, personalization of and flexibility during the intervention were regarded important aspects of (digital) lifestyle support. We learned from interviews with members of the target population that their lifestyle goals are often very specific, person-related, and result-driven on the short term (e.g., losing weight to fit in their favorite jeans rather than to prevent future chronic disease; 63 year old male interview participant “*[…]I can hardly tie my shoelaces. And look, that annoys the hell out of me. But now I've been wearing slippers for 3 months […]so now I'm not annoyed. And soon I'm gonna have to wear my shoes again, and maybe that will cause to flip a switch.”*). Also, members from the target group mentioned that lifestyle advice should be tailored to their personal situation. As we learned that Chinese elderly often perform *tai chi* or square dancing (i.e., low-key dancing groups on public squares) in order to stay active, we included corresponding options to the Chinese mobile application. A comprehensive overview of adaptations made to the mobile application based on input from the PRODEMOS target population can be found in Supplementary Table 2.

### Technical Development

Following the lessons learned, technical development of the UK platform commenced in April 2019. In accordance with the project planning, development in China started in July 2020. Due to differences in hosting requirements between the countries, both platforms were developed and hosted in separate environments in the UK and China. As mentioned previously, both platforms were developed based on the same concepts and requirements, with certain cultural adaptations wherever deemed necessary.

The development of the platforms followed an iterative process, allowing for timely redirection and adaptations. Development was evaluated every other week with the European and Chinese software developers. To bridge the gap between (medical) researchers and technical developers, we used storyboards, containing user-stories, and functional flow block diagrams, mapping all connections between the coach portal and the mobile application. The platform and mobile application were ready for preliminary internal testing by the developers and coordinating research team 5 months after the initial start of development. An overview of the basic functionalities of the PRODEMOS application can be found in Supplementary Figure 1.

### Evaluation and Adaptation Phase

#### Internal Testing

After internal testing by the technical developers, the software was meticulously tested by the coordinating research team to detect potential technical issues, e.g., software bugs. One or more software developers were present during these test sessions to immediately investigate encountered issues and to deliver technical support where needed. After several test cycles, researchers and health coaches from the British and Chinese teams gained access to the mobile application and coach portal to interactively test the system over a longer period of time. The majority of findings concerned software bugs that had to do with the interaction between the mobile application and coach portal. Findings were recorded in a living document. After prioritization on relevance, urgency, and feasibility in collaborative sessions, findings were resolved by the software developers.

#### User Test and Pilot

After internal testing, the platforms were evaluated through thinking aloud sessions and pilot studies. The thinking aloud session provided good insight into the (intuitive) handling of the mobile application by our target population. Findings yielded mostly suggestions to further improve its usability and user-friendliness. Subsequently, the mobile application and portal were tested in a six-week pilot study in both the UK (*n* = 21) and China (*n* = 56). This way, we gained information about frequently used and potentially neglected functionalities and options in the app (e.g., goals were often set by sending chat messages to the coach rather than by using the goal-setting engine; the library was often overlooked).

In the UK, participants indicated the need for more intuitive operationalization of the mobile application with a consistent user interface. Text density and font size needed to be adjusted to better suit the target population. Moreover, log-in procedures were often found to be too complex. In China, as there is already a lot of information available on *WeChat*, participants stressed the need for more in-depth education material.

Coaches in the UK expressed the need to further improve the graphical overview of the progress of participants. Moreover, coaches felt like they would be able to support participants better if they were able to help participants with their goal setting by adjusting certain aspects, such as the evaluation date or the goal target, to make the goals more achievable or relevant. Additionally, coaches in China indicated the need for more extensive instructional information explaining the mobile application and coach portal. A more detailed description of adaptations made to the mobile application and platform based on the evaluation findings is displayed in Supplementary Table 2.

## Discussion

In this paper, we describe the design, development, and piloting of an mHealth portal and mobile application for the prevention of dementia in the context of the PRODEMOS trial, building upon the existing evidence and experience from the HATICE eHealth platform. Based on extensive input from all stakeholders involved, we developed a platform for behavior change for older adults, with adaptations for specific needs from the low SES population in the UK and the general population in China.

For the thorough development of an mHealth platform, many stakeholders from several backgrounds need to be involved, including researchers, healthcare professionals, software developers, and the target population. We believe clear communication is crucial to understand each other's idioms and ways of thinking during development and evaluation. We identified several learning points for open and clear communication between the involved parties. Structural (weekly) meetings stimulated transmission of knowledge and updates on progression. We believe this kept the whole team informed on advancement and allowed timely redirection if necessary. During these meetings, we kept structural documentation on wishes, adaptations and platform errors.

Involving potential end-users in the development process is thought to result in a more appropriate platform design (23–26). To optimally benefit from the feedback of (potential) end-users, we think the timing of these evaluation sessions is of great importance. Early involvement of end-users may be ideal, giving the developers sufficient time to optimally translate feedback into platform development. However, we learned that obtaining specific feedback on platform functionalities in the early stages can be very challenging for potential end-users, given its theoretical and conceptual rather than practical setting. Demonstrating a prototype of the platform, by using clickable designs and wireframes, can make these concepts more tangible, probably increasing the yield of end-user involvement in platform development. User testing of the preliminary functionalities through thinking aloud sessions greatly improved our insights in potential pitfalls of the platform and allowed for early adaptations. Our experience was that the direct presence of software developers during these sessions can benefit the user-centered design process, resulting in more mutual understanding and, ultimately, greater efficiency and quality.

### Limitations

While the evidence-based development of the PRODEMOS portal and mobile application provides exciting opportunities to test the efficacy and implementation of an mHealth intervention in vulnerable populations, we faced several limitations. During focus groups, potential end-users expressed a wish for peer contact and activities to initiate and sustain behavior change. We have investigated the possibilities of incorporating this in our platform, however, concluded that this would yield too many complications regarding organization and privacy regulation. A similar limitation was the integration of external health monitoring devices and other health applications with the PRODEMOS application. Due to the variety and rapidly advancing technologies of smartphones and wearable sensors, we could not ensure continued compatibility of these monitoring tools and decided not to integrate them in our mobile application.

The PRODEMOS mobile application was specifically designed for older, vulnerable populations, integrating a simple, intuitive interface, with written and digital instruction manuals and in-person familiarization with the mobile application, guided by the health coach. However, it is conceivable that part of the target population may not be able to overcome some of the technological challenges involved in using the mobile application. Additionally, to use the application, participants need to have regular and affordable access to the internet. Increasing smartphone possession and usage among older adults suggests that this may be a decreasing barrier (27). Until this barrier is completely omitted, mHealth should be complimentary to alternative methods to facilitate behavior change in older adults. Finally, mHealth is a rapidly advancing field, therefore it is important to appraise the reported findings within the context of this changing landscape of innovation, for example by taking new software features and design trends into account (28).

### Implications for Future Practice and Research

mHealth may recently have become an even more attractive and desirable way to deliver interventions for risk factor management and disease prevention, as the COVID-19 pandemic has highlighted the need for preventive care that can be accessed remotely. Despite the increasing availability of mHealth applications for the prevention of dementia and cardiovascular disease, studies on the development, implementation, and effectiveness of these platforms are scarce. In order to demonstrate the added value of such technologies, there is an urgent need for evidence-based mHealth interventions and high-quality evaluation studies (29). We believe that when developing such digital interventions, early involvement of end-users and other stakeholders will likely aid success and implementation. Moreover, development of a platform that is sustainably used could benefit from consistency of team members and documentation of all steps and decisions taken during each phase of development.

The actual use and usability of the PRODEMOS intervention will be assessed over the coming years in the PRODEMOS trial, with a dual focus on effectiveness and implementation outcomes. If effective, it likely increases the yield of preventive programs in resource-poor settings. If implementable, it will contribute to an improved understanding how such interventions may be successfully provided in the real-world setting.

## Data Availability Statement

The raw data supporting the conclusions of this article will be made available by the authors, without undue reservation.

## Ethics Statement

The studies involving human participants were reviewed and approved by Health Research Authority (HRA) and Health and Care Research Wales (HCRW) and the Medical Ethics Committee of Capital Medical University (CMU), the Medical Ethics Committee of Taishan Medical University (TSMU), the Medical Ethics Committee of the General Hospital of the People's Liberation Army (PLAGH), and the Medical Ethics Committee of Beijing Geriatric Hospital (BGH). The patients/participants provided their written informed consent to participate in this study.

## Author Contributions

MH and EE were responsible for the drafting of the manuscript. MH, EE, and MW were responsible for designing included figures. ER, EC, MH-B, CB, and WeiW were responsible for the study conception. ER, EC, MH-B, MS, SA, CB, NC, JG, HM, WenW, YW, AW, and WeiW were responsible for the design of the trial. MH, EE, MH-B, MW, LB, RB, AG, MM, LS, MS, XY, EC, and ER were involved in platform design and development. All authors were responsible for critically revising the manuscript and approved the final version of the manuscript.

## Funding

This project has received funding from the European Union's Horizon 2020 Research and Innovation Programme under grant agreement No. 779 238 and the National Key R&D Programme of China (2017YFE0118800).

## Conflict of Interest

AG and MM were employed by Philips VitalHealth. LS and XY were employed by Fuzhou Comvee Network &Technology Co. The remaining authors declare that the research was conducted in the absence of any commercial or financial relationships that could be construed as a potential conflict of interest.

## Publisher's Note

All claims expressed in this article are solely those of the authors and do not necessarily represent those of their affiliated organizations, or those of the publisher, the editors and the reviewers. Any product that may be evaluated in this article, or claim that may be made by its manufacturer, is not guaranteed or endorsed by the publisher.
