# Supplementary material for: Design and Development of a Mobile Health (mHealth) Platform for Dementia Prevention in the Prevention of Dementia by Mobile Phone Applications (PRODEMOS) Project
Source: Front Neurol. 2021 Dec 16;12:733878. doi: 10.3389/fneur.2021.733878 (PMC8716458; doi:10.3389/fneur.2021.733878)
Supplement: Supplementary file 1 [file Table_1.DOCX]

**Supplementary table** 1. Evaluation processes during the conceptualization, development and evaluation and adaptation phase

| Target group | N | Date | Method | | Demographics | Recruitment | |
| --- | --- | --- | --- | --- | --- | --- | --- |
| HATICE Evaluation | | | | | | | |
| Former participants of the HATICE study | 6 | Mar  2018 | Structured focus group guided by predefined topic lists, covering: *experiences with HATICE platform, role of the health coach, goal setting and monitoring functionalities, suggested improvements.*  Focus groups were transcribed verbatim; summaries were shared with the research group | | 50% male, mean age 69 years, 2-3 dementia risk factors. | Recruited via health care centre that participated in the HATICE study | |
| Former coaches of HATICE study | 3  3 | Sep 2018  May 2019 | Structured focus group guided by predefined topic lists, covering: *logistics during trial, coaching of participants, use of platform, suggested improvements.*  Focus groups were transcribed verbatim; summaries were shared with the research group | | 100% female, mean age 57 years  100% female,  mean age 58 years | Invited by email, sent to all former HATICE coaches | |
| Potential PRODEMOS end-users † | | | | | | | |
| Interviews with Dutch participants | 19 | 2018-2019 | Semi-structured interviews, guided by a predefined interview guide that was iteratively adapted when necessary. Topics covered included: *experiences and attitudes regarding lifestyle behaviour change and needs for and views on the potential role for mHealth.* Participants were purposively sampled on age, sex, and history of CVD and diabetes.  Interviews were transcribed verbatim, coded by two researchers independently, and thematically analysed. Findings were shared with the research group and will be published. | | 58% male, median age 67 years, 63% history of CVD | Participants were recruited through six general practices in the Netherlands. | |
| Interviews with Chinese participants [21] | 26 | Feb-Dec 2019 | Semi-structured interviews, guided by a predefined interview guide that was iteratively adapted when necessary. Topics covered included: *experiences and attitudes regarding lifestyle behaviour change, needs for coaching, and the use of mHealth.* Participants were purposively sampled on age, sex, living area, and history of CVD and diabetes.  Interviews were transcribed verbatim, coded by two researchers independently, and thematically analysed. Findings were shared with the research group and will be published. | | 50% male,  mean age 70 years,  35% history of CVD | Participants were recruited through seven Chinese hospitals participating in the PRODEMOS study. | |
| Dutch potential end-users with cardiovascular disease | 10 | Feb 2019 | Structured focus group guided by predefined topic lists, covering: *Coaching for lifestyle change, interface of lifestyle application (guided by example screenshots), goal setting, education material.*  Focus groups were transcribed verbatim; summaries were shared with the research group | | 60% male,  mean age 71 years.  All participants had a history of CVD. Two participants did not possess a smartphone. | Recruited via cardiac rehabilitation group | |
| British potential end-users | 2 | April 2019 | Structured interview guided by predefined interview guide, covering: *working with a remote coach, working with predefined goals, education material,* *interface and functionalities of lifestyle application (guided by example screenshots).*  Interviews were transcribed verbatim; summaries were shared with the research group | | 100% female,  mean age 56 years. | Recruited via UK based general practitioner | |
| British potential end-users | 3 | June 2019 | Structured interview guided by predefined interview guide, covering: *use of health applications, goal setting, sustained engagement, monitoring of progress.*  Interviews were transcribed verbatim; summaries were shared with the research group | | 100% female,  aged 40-66 years | Recruited via UK based general practitioner and word-to-mouth | |
| British potential end-users | 5 | Jan 2020 | User test structured by predefined user testing scripts. These scripts covered several scenarios (*contact with coach, setting a new goal, entering a measurement)* that participants had to follow through on the prototype of the app. Usage of the app was observed and evaluated by one researcher and one developer.  User tests were followed up by individual interviews that were guided by the topics covered during user testing. Findings were summarized and shared with the research group | | 40% female, aged 55-75 years | Recruited via participating general practice surgery | |
| British PRODEMOS pilot study participants | 5 | Feb 2020 | Structured focus group guided by predefined topic lists, covering: *Contact with the coach, goal setting, measurement entering, health information, general app usage.*  Focus groups were transcribed verbatim; summaries were shared with the research group | | 40% female, aged 55-75 years | Participants of the PRODEMOS pilot study were invited to participate in a dedicated focus group during the final visit. | |
| Chinese PRODEMOS pilot study participants | 15 | Apr 2021 | Structured interview guided by predefined topic lists covering: *Contact with the health coach, goal setting, measurement entering, health information, general app usage, working on lifestyle improvements using the app*  Interviews were transcribed verbatim; summaries were shared with the research group | | 33% male,  mean age 64 years | Participants of the PRODEMOS pilot study were invited to participate in a dedicated interview during the final visit. | |
| Health coaches and professionals | | | | | | | |
| Experienced community health coaches from Brighton, UK | 4 | Feb 2019 | Structured focus group guided by predefined topic lists, covering:  *working with deprived populations, training of coaches, available health sources, coaching with mHealth.*  Focus groups were transcribed verbatim; summaries were shared with the research group | | 100% female,  multiple years’ experience as health coach in deprived areas. | Recruited via University of Sussex, word-to-mouth | |
| Experienced UK Clinical Research Network health coaches | 2 | June 2019 | Structured focus group guided by predefined topic lists, covering: *Coaching experiences, use of health applications, goal setting.*  Focus groups were transcribed verbatim; summaries were shared with the research group | | 100% female,  multiple years’ experience as health coach in deprived areas. | Recruited via NHS clinical research network | |
| Health care professionals (UK general practitioner and practice nurse) | 2 | June 2019 | Structured interview guided by predefined interview guide, covering: *working with the target population, education of (deprived) patients, monitoring of progress, sustained engagement.*  Interviews were transcribed verbatim; summaries were shared with the research group | | 50% male,  Both multipole years’ experience working in deprived areas. | Recruited via University of Sussex, word-to-mouth | |
| British coaches after PRODEMOS pilot | 2 | Feb 2020 | Structured focus group guided by predefined topic lists, covering: *study logistics, contact with participants, patient management, goal evaluation.*  Focus groups were transcribed verbatim; summaries were shared with the research group | | 100% female | Participating coaches in the PRODEMOS pilot study were invited to participate in a dedicated focus group | |
| Chinese coaches after PRODEMOS pilot | 13 | Apr 2021 | Structured focus group guided by predefined topic lists, covering: *Contact with the coach, goal setting, measurement entering, health information, general app usage.*  Focus groups were transcribed verbatim; summaries were shared with the research group | 23% male,  mean age 33  Differing levels of experience: nurses, health management specialist, under- and postgraduate students majored in clinical medicine with experience | | | Coaches of the PRODEMOS pilot study were invited to participate in a dedicated focus group during the final visit. |

† *Participants were included based on the PRODEMOS study inclusion criteria; aged 55-57 years, living in an area ranked as equal to or less than the lowest third decile of index of multiple deprivation (UK only), possession of smartphone, two or more dementia risk factors, and smartphone literate (being able to send a message).*

*Abbreviations: CVD; cardiovascular disease, UK; United Kingdom,*
