# Supplementary material for: Design and Development of a Mobile Health (mHealth) Platform for Dementia Prevention in the Prevention of Dementia by Mobile Phone Applications (PRODEMOS) Project
Source: Front Neurol. 2021 Dec 16;12:733878. doi: 10.3389/fneur.2021.733878 (PMC8716458; doi:10.3389/fneur.2021.733878)
Supplement: Supplementary file 2 [file Table_2.DOCX]

| **Evaluation finding** | | **Adaptation** |
| --- | --- | --- |
| **Lessons learned from HATICE^a^** | | |
| Participants | Reactive platform use in HATICE | Automatic reminders for engagement with intervention (for example to enter measurements or received chat message) |
|  | Tailored and frequently updated education material | Goal-specific education material flows; new education items (based on the goals set by the participant) are sent to the participant’s library weekly |
|  | Need for more options to tailor the intervention | Intermediate adjustments to goals, for example editing the evaluation date and the goal target |
|  |  | Possibility to tailor the notification frequency per goal  (daily, bi-weekly, weekly) |
|  |  | Pause-functionality, which temporarily stops all notifications for a predefined period of time (e.g. in case of illness) |
|  | Need for more support to use platform | Explanatory video describing the basic platform functionalities (accessible in library) |
| Coaches | Need for better coaching functionalities to create a better overview of participants | Feature to add notes about individual participants |
|  |  | Graphic overviews of participant’s progress |
|  |  | Tailored notifications to alert coaches, for example about newly set goals and achieved milestones. |
|  |  | Interactive goal overview page to help adjust goals from participants |
| **Lessons learned from interviews and focus groups with the PRODEMOS target population** | | |
| Participants | User-friendliness | Wide range of pre-defined goals, including country specific options |
|  |  | Positive wording throughout the application |
|  |  | Carefully selected education items from governmental health institutions, including (peer) videos |
|  |  | Simplified login procedure with 5 (UK^b^)- or 4 (CH^a^)-digit code |
|  |  | Integration with WeChat, using phone number (CH^b^) |
|  | Personalisation | Can add personal notes when setting a goal |
|  |  | Measurement options tailored to be relevant for participants |
|  |  | Tailored education material can be manually sent by coaches |
|  |  | Goal specific education material |
| **Technical development** | | |
| **Evaluation and adaptation based on internal testing, user tests and the pilot study** | | |
| Participants | Accessibility | Decreased text density by using visual substitutes |
|  |  | Increased font size |
|  |  | Changing order on navigation bar to most frequently used |
|  |  | Visualisation of measurement methods |
|  |  | Email address instead of separate username (UK^b^) |
|  |  | Help menu, appraising Frequently Asked Questions (CH^b^) |
|  | More intuitive operationalisation | Button on home screen to directly enter measurements |
|  |  | Equalisation of buttons throughout the application |
|  | More-in-depth material | Adaptation of education material (CH^b^) |
| Coaches | Better patient management | Graphical overview of measurements |
|  |  | Adjustment of goal specifics (date and target of goal) |
|  |  | Voice messages are integrated to communicate with participants (CH^b^) |

**Supplementary table 2.** Overview of main evaluation findings and adaptations to the PRODEMOS platform

**^a^** Development of the PRODEMOS platform built on the Healthy Ageing Through Internet Counselling in the Elderly (HATICE) eHealth platform, which was proven effective for lowering cardiovascular risk of European older adults in a randomized controlled trial (RCT).[13] The coach-supported HATICE platform enabled self-management of cardiovascular risk factors, integrating European guideline recommendations on prevention of cardiovascular disease (CVD) and principles of Bandura’s social-cognitive theory of self-management and behavioural change.

^b^ If an adaptation is country specific this is indicated with (UK) for the United Kingdom and (CH) for China.
