# Supplementary material for: Design and Development of a Mobile Health (mHealth) Platform for Dementia Prevention in the Prevention of Dementia by Mobile Phone Applications (PRODEMOS) Project
Source: Front Neurol. 2021 Dec 16;12:733878. doi: 10.3389/fneur.2021.733878 (PMC8716458; doi:10.3389/fneur.2021.733878)
Supplement: Supplementary file 3 [file Image_1.PDF]

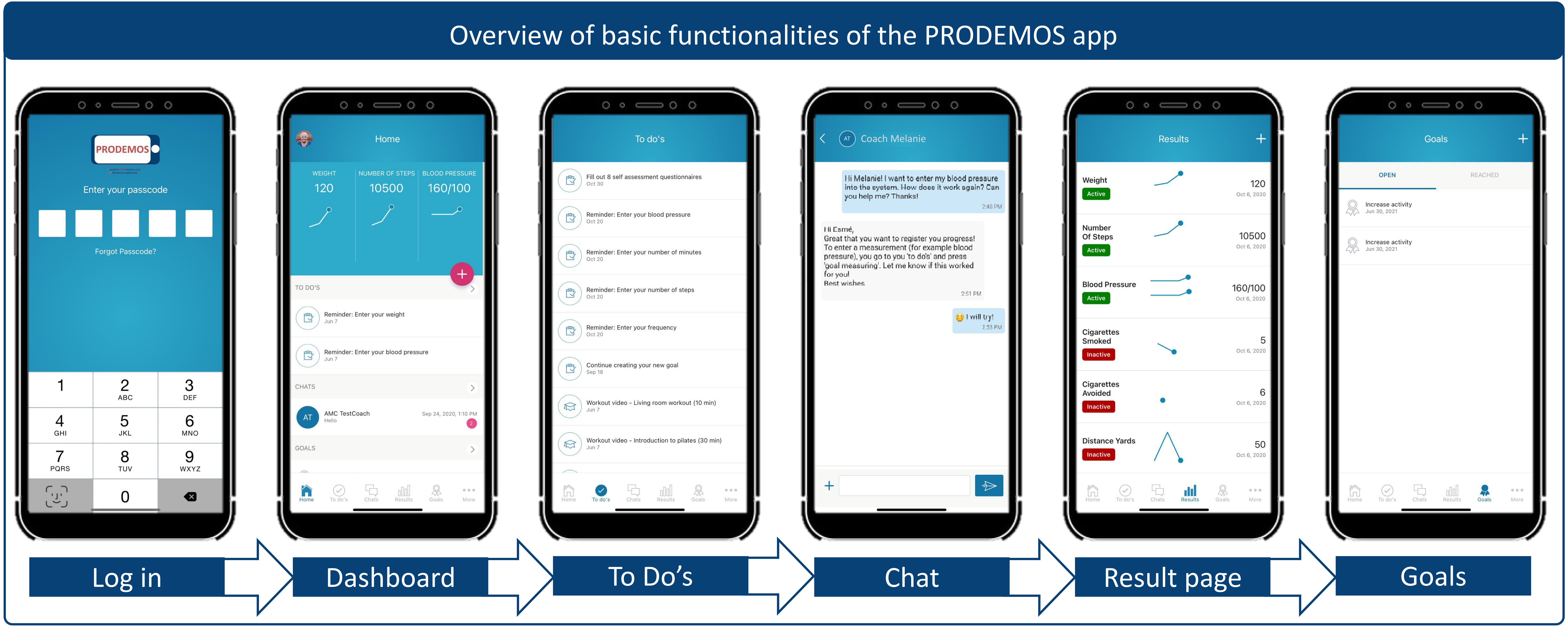

Overview of basic functionalities of the PRODEMOS app
PRODEMOS
Enter your passcode
Forgot Passcode?
Home
WEIGHT
120
NUMBER OF STEPS
10500
BLOOD PRESSURE
160/100
TO DO'S
Reminder: Enter your weight
Reminder: Enter your blood pressure
CHATS
AMC TestCoach
Hello
GOALS
To do's
Fill out 8 self assessment questionnaires
Reminder: Enter your blood pressure
Reminder: Enter your number of minutes
Reminder: Enter your number of steps
Reminder: Enter your frequency
Continue creating your new goal
Workout video - Living room workout (10 min)
Workout video - Introduction to pilates (30 min)
Coach Melanie
Hi Melanie! I want to enter my blood pressure into the system. How does it work again? Can you help me? Thanks!
Hi Esmé, Great that you want to register you progress! To enter a measurement (for example blood pressure), you go to you 'to do's' and press 'goal measuring'. Let me know if this worked for you! Best wishes
I will try!
Results
Weight
Number Of Steps
Blood Pressure
Cigarettes Smoked
Cigarettes Avoided
Distance Yards
Active
Inactive
Goals
OPEN
REACHED
Increase activity
Jun 30, 2021
Log in
Dashboard
To Do's
Chat
Result page
Goals
